# Recombinant adenovirus expressing the haemagglutinin of peste des petits ruminants virus (PPRV) protects goats against challenge with pathogenic virus; a DIVA vaccine for PPR

**DOI:** 10.1186/1297-9716-45-24

**Published:** 2014-02-26

**Authors:** Rebecca Herbert, Jana Baron, Carrie Batten, Michael Baron, Geraldine Taylor

**Affiliations:** 1 The Pirbright Institute, Ash Road, Pirbright, Surrey GU24 0NF, United Kingdom

## Abstract

Peste des petits ruminants virus (PPRV) is a morbillivirus that can cause severe disease in sheep and goats, characterised by pyrexia, pneumo-enteritis, and gastritis. The socio-economic burden of the disease is increasing in underdeveloped countries, with poor livestock keepers being affected the most. Current vaccines consist of cell-culture attenuated strains of PPRV, which induce a similar antibody profile to that induced by natural infection. Generation of a vaccine that enables differentiation of infected from vaccinated animals (DIVA) would benefit PPR control and eradication programmes, particularly in the later stages of an eradication campaign and for countries where the disease is not endemic. In order to create a vaccine that would enable infected animals to be distinguished from vaccinated ones (DIVA vaccine), we have evaluated the immunogenicity of recombinant fowlpox (FP) and replication-defective recombinant human adenovirus 5 (Ad), expressing PPRV F and H proteins, in goats. The Ad constructs induced higher levels of virus-specific and neutralising antibodies, and primed greater numbers of CD8^+^ T cells than the FP-vectored vaccines. Importantly, a single dose of Ad-H, with or without the addition of Ad expressing ovine granulocyte macrophage colony-stimulating factor and/or ovine interleukin-2, not only induced strong antibody and cell-mediated immunity but also completely protected goats against challenge with virulent PPRV, 4 months after vaccination. Replication-defective Ad-H therefore offers the possibility of an effective DIVA vaccine.

## Introduction

Peste des petits ruminants virus (PPRV) causes a devastating disease in goats with mortality rates reaching 70% and higher depending on the virus isolate and health of the animals. The virus is widespread throughout Africa, Asia and the Middle East. Clinical signs of disease include leukopenia, pyrexia, congestion of mucosal surfaces, severe ocular and nasal discharge, necrotic stomatitis, diarrhoea and suppression of the immune system often leading to co-infections. Currently, live attenuated PPRV vaccines are available and can protect animals from subsequent infection. However, these vaccines are not thermostable, requiring a cold chain for delivery to the field which is an added issue, as countries most affected by the disease are hot and often have limited infrastructure. While work is in progress in other labs to improve the thermostability of lyophilised PPRV preparations, development of an intrinsically more thermotolerant vaccine, such as poxvirus- or adenovirus-vectored vaccines would be beneficial. Vaccinated animals produce high levels of neutralizing antibodies against the haemaglutinin (H) and fusion (F) proteins as well as non-neutralizing antibodies against the nucleocapsid protein (N), similar to that seen in animals that have recovered from natural infection [1]. These vaccines do not allow infected-recovered animals to be distinguished from vaccinated animals. A vaccine that allows differentiation of infected from vaccinated animals (DIVA) would be of value in PPRV control programmes as well as a PPRV eradication campaign.

Previous studies have suggested that protective immunity against PPRV could be elicited by expression of just the viral glycoproteins. Recombinant vaccinia virus expressing F and H proteins of rinderpest virus (RPV), which is a close relative of PPRV, protected goats against PPRV challenge, although it did not induce PPRV-specific neutralising antibodies [2]. Similarly, recombinant capripox viruses expressing F and H proteins from RPV [3], or PPRV H or F have been shown to protect goats against PPR [4]. We have sought to evaluate two alternative vectors for expression of the PPRV H and F glycoproteins, fowlpox virus (FP) and replication-defective human adenovirus type 5 (Ad). Recombinant FP-based vaccines have been proven to be effective when used in mammals, despite their inability to replicate in mammalian cells [5,6]. Replication-defective adenovirus vectors have been shown to be a promising platform for delivery of vaccine antigens in a number of species. Although many conventional vaccines are based on induction of protective antibodies, it is clear that, for many pathogens, induction of CD8^+^ T-cell responses are critical for rapid clearance of the pathogen [7]. Vaccination with Ad vectors have been shown to elicit better CD8^+^ T-cell responses compared with poxvirus vectors [8]. The CD8^+^ T-cell response elicited by Ad5 is predominantly an effector memory phenotype [9]. Ad5 induces a CD8^+^ T-cell response with a protracted contraction phase and sustained memory population [10-12]. Ad-based vaccines have shown promise as a single dose vaccine in mice against respiratory syncytial virus [13], *Mycobacterium tuberculosis*[14] and measles virus [15].

If such recombinant viruses are to be useful as field vaccines against PPR, it will be important that they are effective after a single dose, since the main cost in large scale vaccination campaigns is taken up by distribution and administration of vaccines. We have therefore investigated the possible adjuvant properties of virus-vectored delivery of cytokines, granulocyte macrophage colony stimulating factor (GMCSF) and interleukin-2 (IL-2). GMSCF is a cytokine important for recruitment, activation and maturation of antigen-presenting cells [16]. In a number of DNA vaccine studies, GMCSF has been shown to have adjuvant properties [17-21]. Furthermore, GMCSF expressed by recombinant FP has been shown to enhance CTL responses in mice compared to vaccination with antigen and recombinant GMCSF protein [22]. However, other groups have found that GMCSF did not enhance CD8^+^ T-cell or antibody responses [23-25]. IL-2 is involved in activation and recruitment of immune effector cells such as T cells, NK cells, B cells and antigen-presenting cells [26,27]. Co-delivery of IL-2 with DNA vaccination has been shown to enhance antibody responses and increase protection in a variety of animal species [28-30]. Furthermore, IL-2 in combination with GMCSF has been shown to enhance CTL and memory responses [31,32] and act in combination to have an increased adjuvant effect [33,34]. In a number of other animal diseases, addition of IL-2 and GMCSF to vaccines has shown promise as potential adjuvants [35,36]. We have investigated FP and Ad recombinants as vaccine vectors to deliver PPRV surface glycoproteins to small ruminants. The addition of vectors expressing ovine IL-2 and GMCSF was also investigated. We have shown that a single dose of Ad expressing PPRV H glycoprotein is sufficient to protect goats from PPRV challenge and that the addition of IL-2 may contribute to induction of sterile immunity.

## Materials and methods

### Vaccines

Recombinant FP virus and recombinant Ad virus expressing ovine IL-2, ovine GMCSF, PPRV H or PPRV F protein, as well as control adenovirus constructs expressing GFP (Ad-GFP) [37] or an irrelevant antigen (Ag85) (Ad-85) [38] were produced by the Vector Core Facility, Jenner Institute, Oxford, using Fowlpox 9 [39] and E1, E3 deleted human Adenovirus type 5 (Virapower, Life Technologies) vectors. PPRV F and H coding sequences were derived from the attenuated Nigeria75/1 PPRV vaccine strain [40] and have been previously published [41]. The plasmids OvIL-2/pGEM-T-Easy and OvGM-CSF/pGEM-T-Easy were the gift of Gary Entrican, Moredun Institute, Edinburgh. Titres of recombinant FP virus stocks ranged from 2 × 10^8^ to 1 × 10^9^ PFU/mL, and titres of recombinant Ad virus stocks ranged from 1 × 10^10^ to 2 × 10^11^ IU/mL.

### Cells

Human embryonic kidney (HEK 293) cells were obtained from ECACC (European Collection of Cell Cultures, catalogue No: 85120602) and cultured in D-MEM containing 10% foetal calf serum (FCS), 100 units/mL penicillin, 100 units/mL streptomycin and 50 μg/mL Nystatin (DMEM complete). Chicken embryonic fibroblast cells (CEFs) were prepared by the Microbiological Services department at the Pirbright Institute, Compton site, from 9 day-old Rhode Island Red embryos obtained from the poultry production unit at the institute.

### Peptides

PPRV F and H specific peptides were synthesised by Mimotopes, Ltd. Peptides were designed against the whole protein sequence of each protein and consisted of 15mer peptides, overlapping by 10 amino acids. Peptides were dissolved in DMSO.

### Characterisation of the Ad and FP constructs

For analysis of the expression of PPRV proteins, HEK 293 cells were infected with Ad-F, Ad-H or Ad-GMCSF in 6 well plates for several days until cpe was starting to show. Cells were lysed in SDS-PAGE sample buffer and western blot analysis carried out using an anti-PPRV F monoclonal antibody designed in our laboratory or rabbit polyclonal antibody raised to purified PPRV H (the kind gift of Prof M.S. Shaila, Indian Institute of Science, Bangalore). Parallel samples were analysed using Vero-SLAM cells infected with PPRV. For assays of cytokine expression, HEK 293 cells were infected at a multiplicity of infection (MOI) of 10 in 6 well plates. Virus was allowed to adsorb for 2 h before replacing the inoculum with 2 mL of fresh medium and culturing overnight at 37 °C. The tissue culture supernatants (SNs) were harvested from each well and cell lysates (CLs) were prepared by scraping the cells into 500 μL of sterile water. Chicken embryonic fibroblasts (CEF) were infected with recombinant FP at a MOI of 5 in 6 well plates for 2 h before replacing the inoculum with fresh medium and leaving the cells overnight at 37 °C. CLs and SNs were harvested and stored at -20 °C before analysis of the functional activity of IL-2 and GMCSF produced from infected cells.

GMCSF activity was analysed by measuring the ability of SNs and CLs from Ad- or FP-infected cells to induce proliferation of bone marrow cells. Bone marrow cells were prepared from a fresh goat metacarpal bone. Tissue was rotated in PBS containing 5 mM EDTA at room temperature for one hour to extract cells. The cell suspension was passed first through sterile muslin and then a 70 μm cell strainer before centrifugation for 8 min at 500 × *g* at 4 °C to pellet cells. Contaminating red cells were lysed in ammonium chloride lysis buffer (0.8% NH_4_Cl, 0.1 mM EDTA) and the bone marrow cells washed three times in PBS before re-suspending in RPMI/10 containing 10% goat serum (GS), 5 × 10^-5^ M 2-mercaptoethanol, 100 units/mL penicillin, 100 units/mL streptomycin and 50 μg/mL Nystatin (RPMI complete). Duplicate, 2-fold dilutions of CLs or SNs were incubated with 1 × 10^5^ bone marrow cells per microtitre well. Plates were incubated for 6 days at 37 °C with 5% CO_2_ and then labelled overnight with [^3^H]-thymidine. The lymphocyte proliferative responses were expressed as the ratio of cpm in cultures stimulated with CLs or SNs from virus-infected cells to that of cultures stimulated with CLs and SNs from non-infected cells and expressed as a stimulation index (S.I.). The S.I. for CLs and SNs was combined.

IL-2 activity was analysed by measuring the ability of SNs and CLs from Ad- or FP-infected cells to induce the proliferation of peripheral blood lymphocytes. Duplicate, 2-fold dilutions of CLs or SNs were incubated with heparinised goat blood for 6 days before labelling over-night with [^3^H]-thymidine. The lymphocyte proliferative responses were expressed as the ratio of cpm in cultures stimulated with CLs or SNs from virus-infected cells to that of cultures stimulated with CLs and SNs from non-infected cells and expressed as the S.I.

### Goats and experimental design

Male goats, aged between 6 months and 1 year were sourced locally. All were of European breeds, but were of mixed breeds. All animal studies were carried out in accordance with UK Home Office regulations and under the supervision of the local Ethical Committee. Animals were vaccinated intra-muscularly in the left shoulder with vaccine made up to 1 mL with sterile PBS. The doses of recombinant virus vectors used to vaccinate goats were similar to those that had previously been used in man and goats [39,42].

#### Experiment 1

One animal was vaccinated with a mixture of 1 × 10^9^ IU Ad-F and 1 × 10^9^ IU Ad-H and the other goat was vaccinated with a mixture of 1 × 10^8^ PFU FP-F and 1 × 10^8^ PFU FP-H. Blood was taken weekly for sera and preparation of peripheral blood mononuclear cells (PBMC). Animals were given an homologous boost, 5 weeks later, and were killed 8 weeks post vaccination by intravenous pentobarbitone overdose. At post mortem examination, pre-scapular lymph nodes (PLN) were removed for analysis of H- and F-specific T-cell responses.

#### Experiment 2

Four goats were vaccinated with a mixture of 1 × 10^9^ IU Ad-F and 1 × 10^9^ IFU Ad-H and four goats were vaccinated with a mixture of 1 × 10^8^ PFU FP-F and 1 × 10^8^ PFU FP-H. Two animals from each group were also vaccinated with a mixture of 1 × 10^9^ IU Ad-IL-2 and 1 × 10^9^ IU Ad-GMCSF or a mixture of 1 × 10^8^ PFU FP-IL-2 and 1 × 10^8^ PFU FP-GMCSF respectively. Blood was taken weekly for sera and preparation of PBMC, and animals were killed 12 weeks post vaccination by intravenous pentobarbitone overdose. At post mortem examination, PLN were removed.

#### Experiment 3

Goats were vaccinated as shown in Table 1 with a total of 3 × 10^9^ IU of recombinant Ad per animal. Blood was taken weekly for sera and preparation of PBMC. Animals were challenged intranasally 15 weeks after vaccination with 1 × 10^5^ TCID_50_ of pathogenic PPRV (Ivory Coast/89 isolate). The rectal temperature was measured daily, and an average of the values from day -3 to day 0 of challenge were taken as the basal body temperature for each animal. Clinical assessments were carried out daily and animals scored on a scale from 0 to 2 or 3, based on the severity of ocular, oral and nasal congestion and discharge as well as signs of apathy, anorexia, diarrhoea and ulceration in the mouth, as shown in Table 2. The scores were combined to give an overall clinical score for each day post challenge (pc). Nasal, oral and ocular swabs were taken on day 0, 4 to 8, 11 and 14 pc for analysis of PPRV by quantitative RT-PCR (see below). Nasal swabs taken on day 4, 6, 7 and 10 were also analysed for virus by isolation/titration (VI). Blood was taken in EDTA on days 0, 4, 7, 11 and 14 pc for analysis of white cell counts and viraemia by RT-PCR. Heparinised blood was taken at day 6 pc for preparation of PBMC. Animals were killed by intravenous pentobarbitone overdose either at day 14 pc or earlier if they had reached the humane end point determined by the Home Office project licence.

**Table 1:** Vaccine groups for challenge study

**Group**	**No goats**	**Ad-GFP**	**Ad-H**	**Ad-IL-2**	**Ad-GMCSF**
A	4	1 × 10^9^ IU	-	1 × 10^9^ IU	1 × 10^9^ IU
B	3	2 × 10^9^ IU	1 × 10^9^ IU	-	-
C	3	1 × 10^9^ IU	1 × 10^9^ IU	1 × 10^9^ IU	-
D	3	1 × 10^9^ IU	1 × 10^9^ IU	-	1 × 10^9^ IU
E	3	-	1 × 10^9^ IU	1 × 10^9^ IU	1 × 10^9^ IU

16 outbred, European, male goats, 9 to 12 months of age were vaccinated intra-muscularly with a total of 3 × 10^9^ IU of recombinant adenovirus per animal in a final volume of 1 mL PBS.

**Table 2:** Clinical scores

**Clinical signs**	**0**	**1**	**2**	**3**
**Temperature**	**≤ Initial + 0.1**	**Initial + 0.1 to ≤ initial + 2**	**> Iinitial + 2**	
Nasal signs	None	Congestion	Discharge	
Ocular signs	None	Congestion	Discharge	
Oral signs	None	Congestion gums/papillae	1 or 2 vesicles in gums	Necrotic vesicle(s)
Diarrhoea	None	Diarrhoea	Bloody diarrhoea	
Respiration	Normal	Coughing	Noisy breathing	
Behaviour	Normal	Apathetic	Recumbent	

Nasal swabs were washed in medium (DMEM containing 10% FCS, 100 units/mL penicillin, 100 units/mL streptomycin) to extract virus from the swabs and then serial dilutions were plated with VDS cells in at least quadruplicate, starting with a 1 in 10 dilution, for determination of virus titre by TCID_50_. Alternatively, a 1 in 6 dilution of the sample was used to inoculate VDS cells for virus isolation (VI). Infected cells were cultured for up to 7 days at 37 °C with 5% CO_2_. Wells were scored for the presence of cpe.

Blood samples and swabs taken after PPRV challenge were analysed by reverse transcription-real time PCR [43] to determine the level of viraemia and virus in nasal secretions. RNA was extracted from EDTA blood or swabs, reverse transcribed and amplified with SuperScript® III Platinum® One-Step qRT-PCR w/ROX Kit, (Invitrogen™). Primer 5’-3’sequences were, PPRVFOR:- AGAGTTCAATATGTTRTTAGCCTCCAT;

PPRVREV:- TTCCCCARTCACTCTYCTTTGT;

probe: *FAM*-CACCGGAYACKGCAGCTGACTCAGAA – *Tamra.*

Primers were used at 10 pmol/μL and the probe at 5 pmol/μL. The thermal profile was 50 °C for 30 min, 95 °C for 10 min, then 45 cycles of 95 °C for 15 s followed by 60 °C for 1 min.

### Isolation of PBMCs and lymph node cells

PBMC were prepared from heparinised venous blood by centrifugation at 1200 *g* over Histopaque 1083 (Sigma-Aldrich, Inc.) for 45 min at 20 °C. Cells were washed three times in PBS before re-suspension in RPMI/10 complete medium. Pieces of lymph node were pushed gently through a sterile, metal tea strainer into a petri dish containing PBS, 200 units/mL penicillin, 200 units/mL streptomycin and 100 μg/mL Nystatin. The cell suspension in PBS was further filtered through a 100 μm cell strainer, and cells were purified on histopaque gradients as described above for PBMCs and then cultured in RPMI/10 complete medium. Spare cells were also frozen in FCS with 10% DMSO.

### Serology

Serum antibodies specific for the PPRV H glycoprotein were analysed by competition ELISA (cELISA) using a PPR Antibody ELISA kit (BDSL). Antibodies specific for the PPRV N protein were detected by cELISA using the ID Screen PPR Competition kit (ID Vet).

PPRV neutralising antibodies were analysed essentially as described in the OIE manual, chapter 2.7.11, section 3a. Sera was heat inactivated at 56 °C for 30 min and serially diluted two-fold in quadruplicate. PPRV Nigeria 75/1 was diluted in media to give 100-150 infectious units/50 μL and incubated with the diluted serum at 37 °C in 5% CO_2_ for one hour. Vero-dog-SLAM (VDS) cells were added to the virus/serum mixture at 1 × 10^5^ cells per microtitre well. Plates were incubated for one week and then scored for the presence or absence of cytopathic effect. The neutralising titre was the reciprocal of the highest dilution that completely blocked CPE in 50% of infected wells. Neutralising titres were calculated using the Spearman-Kärber equation to determine TCID_50_.

### Lymphocyte assays

PBMCs were stimulated with either peptide pools, at final concentration of 10 μg/mL, or with Ad or FP viruses. Stimulation with Ad was at a MOI of 100 virus particles and FP at a MOI of 0.1 PFU. Concanavalin A was used as a positive control at a final concentration of 25 μg/mL. All assays were performed in triplicate. The lymphocyte proliferative responses were expressed as the ratio of counts incorporated in cultures stimulated with peptide to counts incorporated in cultures with media containing DMSO at the same concentration as the peptides, or the ratio of counts in cultures stimulated with Ad or FP expressing PPRV antigens to counts in cells stimulated with a corresponding virus expressing an irrelevant antigen. Data is expressed as the S.I., and an S.I. greater than 5 was considered to be significant.

PBMCs and lymphocytes from the PLN were stimulated with peptide pools, or Ad or FP viruses as described above for 24 h and brefeldin A was added for the last 4 h of culture. Live cells were stained using live/dead aqua (Molecular probes®), and monoclonal antibodies (mAbs) to surface markers CD4 conjugated to allophycocanin (APC) (clone 44.38, MCA2213A647 from Serotec) and CD8 conjugated to R-Phycoerythrin (RPE) (clone CC63, MCA837 from Serotec). Cells were permeabilised with BD FACS permeabilisation buffer and stained with mAb to IFNγ (clone CC327, MCA2334 from Serotec). Cells were analysed using the LSR Fortessa (BD Biosciences). The DIVA software was used to acquire the data and FCS Express 3 (De Novo Software) or FlowJo (Tree Star Inc.) used for analysis. The PPRV-specific response was calculated as the percentage of IFNγ^+^ -producing cells after stimulation with Ad or FP constructs expressing the PPRV protein minus the percentage of such cells after stimulation with Ad or FP expressing an irrelevant antigen.

At day 0, 4 and 7 pc with PPRV, heparinised blood was stained with antibodies for surface markers CD4, CD8, using antibodies described above, annexin V antibody conjugated to FITC (Aposcreen kit, SouthernBiotech), CD14 (clone CCG33) antibody conjugated to R-PE and antibody to WC1, a marker for γδ T cells (clone 197), conjugated to APC using a Zenon® kit (Life Technologies). Red blood cells were lysed using BD FACS lysis solution. Cells were fixed in 4% PFA and permeabilised as above. Intracellular virus was detected using the anti-PPRV H monoclonal antibody C77 conjugated to Alexa Fluor-405 using a Zenon® kit (Life Technologies).

## Results

### Characterisation of Ad and FP vaccine vectors expressing PPR glycoproteins, ovine IL-2 or ovine GMCSF

**Figure 1:**
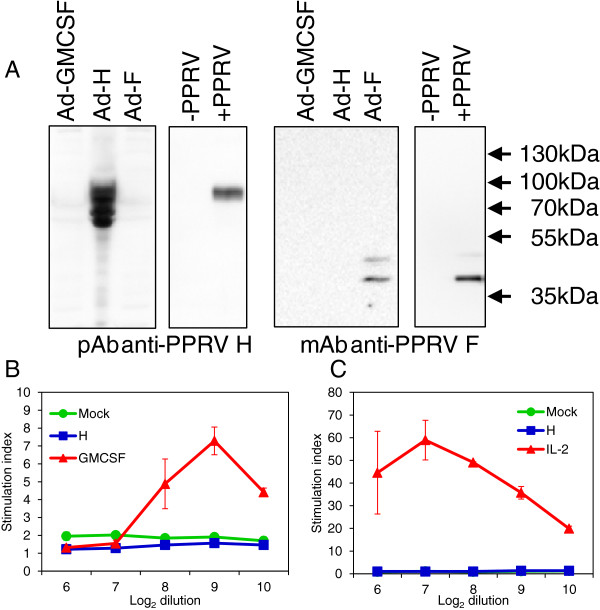
**Expression of PPRV glycoproteins and functionally active ovine IL-2 and GMCSF, in vitro. (A)** HEK 293 cells were infected with Ad-F, Ad-H or Ad-GMCSF, and Vero-SLAM cells were infected with PPRV or mock infected. Total cell lysates were analysed by Western blot using an anti-PPRV F monoclonal antibody or polyclonal rabbit anti-PPRV H serum as indicated. **(B, C)** HEK 293 cells were infected with Ad-IL-2, Ad-GMCSF or Ad-H, or mock infected overnight and cell lysates (CLs) and supernatants (SNs) were harvested. The biological activity of GMCSF in CLs and SNs combined from Ad-GMCSF, Ad-H or mock-infected cells was determined by analysis of proliferation of goat bone marrow cells. The biological activity of IL-2 in SNs from Ad-IL-2, Ad-H or mock-infected cells was determined by analysis of proliferation of goat blood lymphocytes. Proliferation was measured by ^3^H-thymidine incorporation after 7 days incubation and expressed as the mean stimulation index ± S.D. of triplicate wells.

Recombinant viruses were analysed to confirm expression of the PPRV F and H proteins and expression of biologically active IL-2 and GMCSF. CEF cells were infected with FP expressing F or H and the expressed proteins were visualised by confocal microscopy using H- and F-specific mAbs. Clear, specific staining was observed in cells infected with FP-F and FP-H (data not shown). For the Ad recombinants, lysates of HEK 293 cells infected with Ad-F, Ad-H and Ad-GMCSF (as negative control) were analysed by Western blotting to detect the expression of the PPRV proteins (Figure 1A). Bands corresponding to PPRV F (≈ 46 kDa) or H (≈ 80 kDa) proteins were detected, although the H protein appeared to be susceptible to partial degradation in the HEK293 cells when expressed on its own (Figure 1A).

The production of biologically active GMCSF by the recombinant vaccine vectors was confirmed by the finding that CLs and SNs from cells infected with Ad-GMCSF (Figure 1B) or FP-GMSCF (data not shown), but not from mock-infected, Ad-H or FP-H-infected cells, induced the proliferation of goat bone marrow cells. The production of biologically active IL-2 was confirmed by the finding that SNs from Ad-IL-2 (Figure 1C) or FP-IL-2-infected cells (data not shown), but not mock-, Ad-H or FP-H infected cells induced proliferation of goat lymphocytes.

### Immune responses induced in goats vaccinated with Ad or FP expressing PPRV glycoproteins

**Figure 2:**
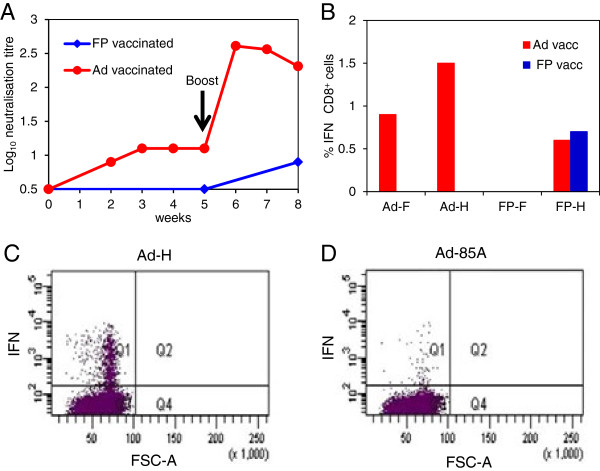
**Immune responses induced in goats vaccinated with Ad or FP expressing PPRV glycoproteins.** One goat was vaccinated intra-muscularly with a mixture of 1 × 10^9^ IU of Ad-H and 1 × 10^9^ IU Ad-F and another goat was vaccinated intra-muscularly with a mixture of 1 × 10^8^ pfu of FP-H and 1 × 10^8^ pfu FP-F. Goats were boosted intra-muscularly with the same doses of the respective vaccines, 5 weeks later. **(A)** Neutralising antibody titres were determined by 50% plaque reduction assay. **(B)** At post mortem examination, 9 weeks after vaccination, F- and H-specific T-cell responses in prescapular lymph nodes were analysed by flow cytometry of lymphocytes stimulated with Ad-H, Ad-F, FP-H, FP-F or control Ad and FP expressing an irrelevant antigen. Cells were stained for CD8 and intracellular IFNγ. Results are expressed as the percentage of CD8^+^ IFNγ^+^ T-cells following stimulation with virus vector expressing F or H minus percentage following stimulation with control virus vectors. Dot plot showing CD8^+^ IFNγ^+^ T-cells from the prescapular lymph node from the Ad-vaccinated goat after stimulation with Ad-H **(C)** and after stimulation with Ad expressing an irrelevant antigen **(D)**.

In order to determine the ability of FP or Ad constructs to elicit PPRV-specific immune responses in goats, preliminary experiments were carried out using 2 animals (experiment 1). The animal vaccinated with Ad-F plus Ad-H showed an increase in PPRV-neutralising antibody titre from week 1 to week 3 post vaccination after which the antibody levels plateaued until boosting at week 5 (Figure 2A). After boosting, the neutralising antibodies increased rapidly. In contrast, neutralising antibodies were not detected in the FP-vaccinated goat prior to boosting, and even after boosting antibody levels were low (Figure 2A). Lymphocyte proliferation and IFNγ ELISpot assays were carried out using PBMC re-stimulated with Ad or FP expressing H, F or an irrelevant antigen. However, the data were inconclusive. At post mortem examination, 3 weeks after boosting, PLN lymphocytes were stimulated with Ad-F, Ad-H (Figure 2C), control AdV, Ad-85 (Figure 2D), FP-F, FP-H or FP control virus, and analysed by flow cytometry to identify IFNγ-producing CD4^+^ and CD8^+^ T-cells. The percentage of PPRV F/H-specific IFNγ-producing CD8^+^ cells in the Ad-vaccinated goat was much higher than in the FP-vaccinated animal (Figure 2B). PPRV-specific IFNγ-producing CD4^+^ cells were not detected in PLNs from either the Ad- or FP-vaccinated goat. Although only one goat was vaccinated with Ad or FP virus vectors, it was clear that vaccination induced an immune response to the expressed PPRV F and H proteins, and so a larger study was carried out to compare single doses of Ad and FP, and to examine the ability of virus-vectored administration of GMCSF and IL-2 to enhance the immune response.

### Effect of virus-vectored ovine IL-2 and GMCSF on immune responses induced in goats vaccinated with Ad or FP constructs expressing PPRV glycoproteins

**Figure 3:**
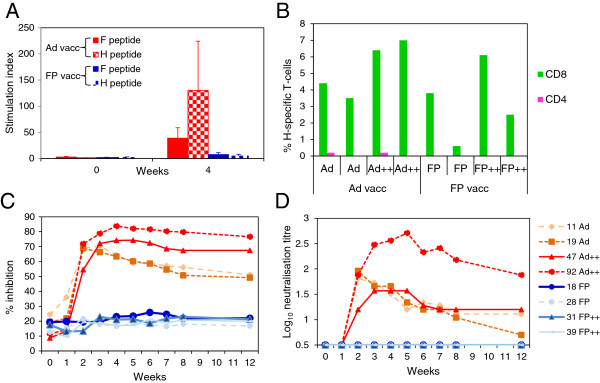
**Effect of virus-vectored ovine IL-2 and GMCSF on immune responses induced in goats vaccinated with Ad or FP expressing PPRV glycoproteins.** Groups of two goats were vaccinated intra-muscularly with a mixture of 1 × 10^9^ IU of Ad-F and 1 × 10^9^ IU of Ad-H with or without the addition of 1 × 10^9^ IU of Ad-IL-2 and 1 × 10^9^ IU of Ad-GMCSF (Ad++), or with a mixture of 1 × 10^8^ pfu FP-F and 1 × 10^8^ pfu FP-H, with or without the addition of 1 × 10^8^ pfu FP-IL-2 and 1 × 10^8^ pfu FP-GMCSF (FP++). **(A)** Priming of F- and H-specific T-cells was determined by proliferation of PBMCs stimulated, in vitro, with peptide pools representing the PPRV F and H proteins. Results are expressed as the mean stimulation index ± S.E for all the Ad-vaccinated or all the FP-vaccinated animals. **(B)** At post mortem examination, 12 weeks after vaccination, H-specific T-cells in prescapular lymph nodes were analysed by flow cytometry of lymphocytes stimulated, in vitro, with H peptides. Results are expressed as % CD4^+^ IFNγ^+^ and CD8^+^ IFNγ^+^ from each animal. **(C)** H-specific serum antibody responses were determined by competition ELISA and expressed as % inhibition of the binding of an H-specific monoclonal antibody to PPRV. **(D)** Virus neutralising antibodies were analysed as described in methods and expressed as Log_10_ neutralising titre.

In order to confirm that Ad vaccination induced a better immune response than FP vaccination and to analyse the adjuvant effects of virus-vectored GMCSF and IL-2, groups of two goats were vaccinated as described in Materials and Methods for experiment 2. Lymphocyte proliferation in response to PPRV H or F proteins at week 0 and 4 post vaccination was analysed following stimulation with pools of PPRV F or H peptides. The responses have been combined for all the Ad-vaccinated animals and for all the FP-vaccinated animals as there was no significant effect of virus-vectored IL-2 plus GMCSF on the lymphocyte proliferative response. At 4 weeks post vaccination, F- and H-specific T-cell responses were only detected in the Ad-vaccinated animals (Figure 3A). In order to further characterise the T-cell mediated immune response induced by the vaccines, PLN lymphocytes, obtained 12 weeks after vaccination, were stimulated with H peptide pools and analysed by flow cytometry. H-specific IFNγ-producing CD8^+^ T-cells were detected in all Ad-vaccinated goats, and responses were slightly greater in the animals also vaccinated with virus-vectored IL-2 and GMCSF (Figure 3B). A low level of H-specific CD4^+^ IFNγ^+^ cells were detected in PLN from 2 out of 4 Ad-vaccinated goats (Figure 3B). H-specific CD8^+^ IFNγ^+^ cells were also detected in PLN from all FP-vaccinated goats. However, the frequency of CD8^+^ IFNγ^+^ cells in 2 of the goats was lower than that seen in the Ad-vaccinated animals (Figure 3B). H-specific CD4^+^ IFNγ^+^ cells were not detected in PLN from FP-vaccinated animals.

From week 2 post-vaccination to week 12, sera from Ad-vaccinated animals showed a significantly greater inhibition of binding of an H-specific mAb to PPRV antigen than sera from FP-vaccinated animals (*p* < 0.0001; 2-way ANOVA) (Figure 3C). Furthermore, the average percentage inhibition by sera from the Ad-vaccinated animals that had also been given Ad-IL-2 and Ad-GMCSF, was significantly greater than the average of the two goats vaccinated with Ad-F and Ad-H only (*p* < 0.05), from week 4 post vaccination to week 12. PPRV-specific neutralising antibodies were not detected in any FP-vaccinated animals (Figure 3D). In contrast, all Ad-vaccinated animals developed neutralising antibodies from week 2 post vaccination, which reached a peak between 2 to 5 weeks after vaccination, and then gradually declined, although 3 out of 4 goats still had neutralising antibody titres > log_10_ 1.0 at 12 weeks after a single vaccination with Ad-vectored vaccines. One goat, which was also vaccinated with Ad-IL-2 plus Ad-GMCSF, had very high levels of neutralising antibodies which peaked 5 weeks after vaccination and remained high at 12 weeks post vaccination.

### Adenovirus vaccination protects against challenge with PPRV

**Figure 4:**
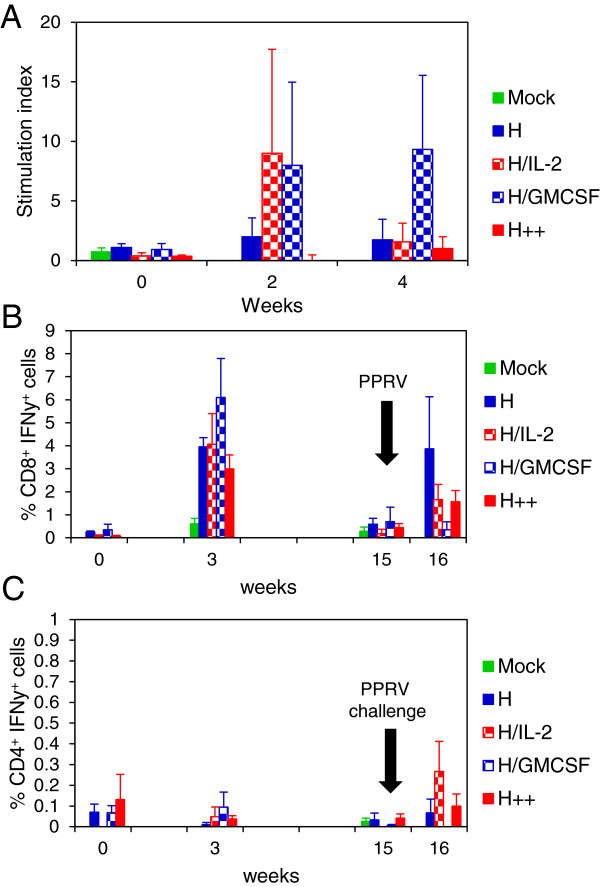
**Effect of virus-vectored ovine IL-2 and/or GMCSF on T-cell responses induced in goats vaccinated with Ad expressing the H protein of PPRV.** Goats were vaccinated intra-muscularly with 1 × 10^9^ IU of each Ad virus as described in Table 1 and were challenged intranasally with 1 × 10^5^ TCID_50_ of pathogenic PPRV, Ivory Coast/89, 15 weeks later. PBMCs were stimulated with H peptides and H-specific T-cell proliferative responses were measured by ^3^H-thymidine incorporation **(A)**; and H-specific CD8^+^ IFNγ^+^**(B)** and CD4^+^ IFNγ^+^**(C)** cells were analysed by flow cytometry. Results are expressed as the mean stimulation index or mean % IFNγ-containing cells ± S.E. of 3 to 4 goats.

Since the major T-cell responses in the Ad-vaccinated animals appeared to be directed to the H protein, and neutralising antibodies are mainly induced by the H protein [1], we tested the ability of this construct to protect against challenge with virulent PPRV. Goats were vaccinated with recombinant Ad viruses as described in Table 1 (experiment 3), and challenged intranasally with pathogenic PPRV (Ivory Coast/89 isolate), 15 weeks later. Following vaccination and after challenge, H-specific T-cell proliferation responses were analysed after stimulation of PBMCs with H protein peptides. At 2 weeks post vaccination, H peptide-specific T-cell proliferative responses were observed in goats vaccinated with Ad-H + Ad-IL-2 and animals vaccinated with Ad-H + Ad-GMCSF, but not in goats in the other vaccine groups (Figure 4A). At 4 weeks post vaccination, H peptide-specific T-cell proliferative responses were only seen in animals vaccinated with Ad-H + Ad-GMCSF. There was considerable variation between animals as indicated by the error bars.

PPRV H-specific IFNγ-producing CD8^+^ cells were detected in all Ad-vaccinated goats 3 weeks after vaccination and the proportion of these cells was highest in animals also vaccinated with Ad-GMCSF (Figure 4B). The proportion of H-specific CD8^+^ IFNγ^+^ cells at the time of PPRV challenge (week 15) was low and similar to that seen prior to vaccination. However one week after challenge, an increase in H-specific IFNγ producing CD8^+^ cells was observed in all the vaccinated animals except the Ad-H + Ad-GMCSF group. In contrast, H-specific IFNγ-producing CD4^+^ T-cells were seen only at very low levels throughout the study, although there was a slight increase in the proportion of CD4^+^ IFNγ^+^ cells in Ad-H + Ad-IL-2 vaccinated animals one week after challenge (Figure 4C). As seen previously, Ad vaccination was more effective at priming CD8^+^ cells than CD4^+^ cells.

**Figure 5:**
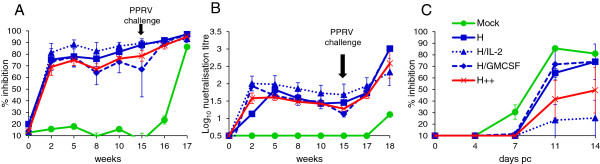
**Effect of virus-vectored ovine IL-2 and/or GMCSF on serum antibody responses induced in goats vaccinated with Ad expressing the H protein of PPRV.** Goats were vaccinated intra-muscularly with 1 × 10^9^ IU of each Ad virus as described in Table 1 and were challenged intranasally with 1 × 10^5^ TCID_50_ of pathogenic PPRV, Ivory Coast/89, 15 weeks later. Mock refers to group A of Table 1 (Ad-GFP + Ad-IL-2 + Ad-GMCSF), while H++ refers to group E of Table 1 (Ad-H + Ad-IL-2 + Ad-GMCSF). **(A)** H-specific serum antibody responses were analysed by competition ELISA and expressed as mean % inhibition of the binding of an H-specific monoclonal antibody to PPRV ± S.D. **(B)** Virus neutralising antibody titres were determined by 50% plaque reduction assay and expressed as log_10_ neutralising titre ± S.D. **(C)** The N-specific serum antibody response was analysed by ELISA and the post-challenge (pc) response is expressed as mean % inhibition of the binding of a PPRV N-specific monoclonal antibody ± S.E.

By week 2 post vaccination, H-specific serum antibodies were detected by cELISA in all the Ad5-H vaccinated animals (Figure 5A). There was little difference in the ability of sera from the Ad-H vaccinated groups to inhibit the binding of the H-specific mAb to PPRV. After challenge, H-specific serum antibodies increased rapidly in all animals. PPRV neutralising antibodies were detected in sera from all the Ad-H vaccinated animals from week 2 post vaccination and remained high until challenge (Figure 5B). After challenge, there was a corresponding sharp increase in the level of neutralising antibodies in all Ad-H and control vaccinated animals. H-specific (cELISA-reactive) and neutralising antibodies were not detected in the control vaccine group prior to challenge. The vaccine group which received Ad-H and Ad-IL-2 had slightly higher levels of neutralising antibodies and H-specific antibodies than the other vaccine groups. However, the differences were small.

In order to assess the potential of Ad-H as a DIVA vaccine, we analysed the development of antibodies to the PPRV nucleocapsid (N) protein in vaccinated goats before and after challenge with PPRV. As expected, none of the animals had N-specific antibodies before challenge. However, N-specific antibodies were detected from day 7 pc in the controls (Figure 5C). N-specific antibodies were slower to develop in the Ad5-H vaccine groups and were first detected at days 11 and 14 pc. Analysis of the level of N-specific antibodies suggested that they were significantly lower in goats vaccinated with Ad-H plus Ad-IL-2 (*P* < 0.001). Ad-GMCSF did not appear to have an effect on induction of N-specific antibodies after challenge.

**Figure 6:**
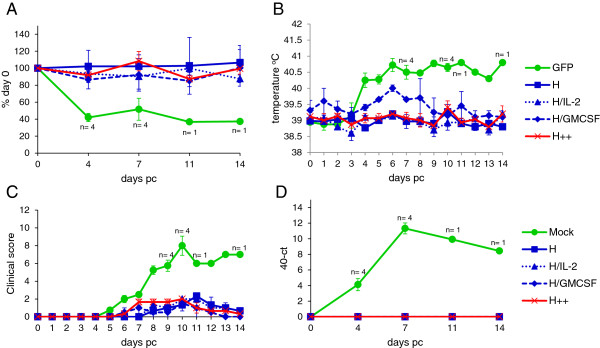
**Vaccination with Ad-H protects against PPRV challenge.** Goats were vaccinated as described in Table 1 and challenged with PPRV as described in Figure 4. Mock refers to group A of Table 1 (GFP + IL-2 + GMCSF), while H++ refers to group E of Table 1 (H + IL-2 + GMCSF). **(A)** Changes in the number of blood leukocytes were determined daily after challenge and results are expressed as a % of day 0 value ± S.E. **(B)** Rectal temperatures were obtained daily and expressed mean body temperature ± S.E. **(C)** Signs of clinical disease were assessed daily and animals given a score based on the severity of congestion in the mucosa of the eyes, nose and gums, ocular and nasal discharge, temperature, diarrhoea, necrotic stomatitis and general malaise. Results are expressed as the mean score ± S.E. **(D)** Viremia was determined by qPCR and expressed as the mean 40 - ct value ± S.E.

Vaccination with Ad-H induced complete protection against challenge with virulent PPRV. Following PPRV challenge, there was a rapid decline in blood leucocytes in the control goats, characteristic of PPRV infection, which was not observed in any of the Ad-H vaccinated groups (Figure 6A). White cell counts did not recover in the surviving control animal before the end of the study. All animals in the control vaccine group developed an increase in temperature by day 4 pc (Figure 6B). A slightly elevated body temperature was seen in goats vaccinated with Ad-H + Ad-GMCSF from day 5 to day 8 pc. However, the temperatures of these animals varied even prior to challenge, and the peak temperature (at day 6) was not significantly higher than that seen at day 1 pc. The body temperatures of the other Ad-H vaccinated groups remained fairly constant following PPRV challenge (Figure 6B). The goats in the control group were the only animals which exhibited any significant signs of clinical disease, and 3 out of 4 animals had to be euthanized prior to the end of the experiment as they had reached the permitted humane end-point for the study (Figure 6C). A number of Ad-H vaccinated goats had a slight redness around the nose and eyes but this may have been due to other factors not associated with PPRV challenge, such as swabbing. Virus RNA was detected in blood (Figure 6D) and virus was isolated from the nasopharynx (Table 3) and ocular swabs (data not shown) from all control animals at day 7 pc. In addition, virus was isolated from nasal swabs on day 6 and 10 pc in some of the control animals (data not shown). In contrast, viral genome was not detected in the blood, and live virus was not isolated from nasopharyngeal swabs of any of the Ad-H vaccinated animals (Figure 6D and Table 3).

**Table 3:** PPRV in the nasopharynx 7 days post challenge (pc)

**Vaccine**^**a**^	**Mean PPRV titre in nasopharynx (log**_**10**_**TCDI**_**50**_**/mL)**^**b**^
Ad-GFP + Ad-IL-2 + Ad-GMCSF	3.88 +/- 0.84 (4/4)^c^
Ad-H + Ad-GFP	< 0.1 (0/3)
Ad-H + Ad-IL-2	< 0.1 (0/3)
Ad-H + Ad-GMCSF	< 0.1 (0/3)
Ad-H + Ad-IL-2 + Ad-GMCSF	< 0.1 (0/3)

^a^Goats were vaccinated as described in Table 1 and challenged with PPRV as described in Figure 3.

^b^Titres of PPRV in nasal swabs on day 7 pc were analysed on SLAM-Vero cells and results expressed as the mean log_10_ TCID_50_/mL.

^c^Number of goats from which PPRV was isolated/total on day 7 pc.

**Figure 7:**
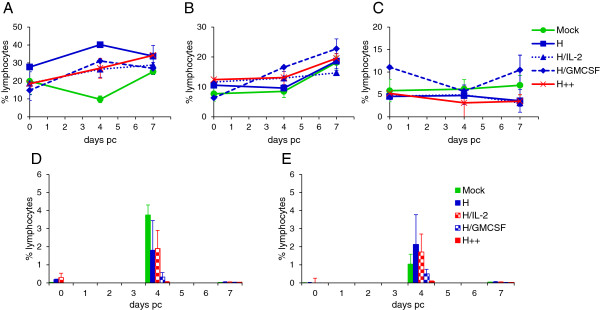
**Effect of virus-vectored ovine IL-2 and/or GMCSF on CD4 and CD8 T-cells and CD14**^**+**^**monocyte/macrophages in vaccinated goats, following PPRV challenge.** Goats were vaccinated and challenged as described in Figure 4. Blood was stained with anti-CD4, anti-CD8 and anti-CD14 monoclonal antibodies directly conjugated to fluorochromes. Cells were also stained for intracellular virus using fluorochrome-labelled anti-PPRV H, C77, monoclonal antibody. Results are expressed as the mean percentage ± SD of CD4^+^ lymphocytes **(A)**, CD8^+^ lymphocytes **(B)** and CD14^+^ cells **(C)** in peripheral blood. The percentage of CD4^+^**(D)** and CD8^+^**(E)** lymphocytes with intracellular staining with C77. Data is presented as the mean percentage ± S.E., 0, 4 and 7 days after PPRV challenge.

The effect of PPRV infection of the proportion of lymphocyte subsets in peripheral blood was also analysed by flow cytometry. The proportion of live CD4^+^ cells decreased at day 4 pc in the control group by about 10% (Figure 7A). This decrease was associated with an increase in dead CD4^+^ cells at this time point (data not shown). At day 7 pc, there was an increase in live CD8^+^ cells (Figure 7B) and also dead CD8^+^ cells (data not shown). There was no change in the percentage of live γδ-T cells (data not shown) or CD14^+^ monocyte/macrophage cells (Figure 7C). Intracellular PPRV H protein was only detected in live CD4^+^ (Figure 7D) and CD8^+^ (Figure 7E) cells at day 4 pc. Intracellular H protein was detected in 4% of CD4^+^ cells from control vaccinated animals, which was greater than that detected in Ad-H-vaccinated goats. However, there was considerable variation in the proportion of CD4^+^ cells with intracellular H protein in these animals. The proportion of CD8^+^ cells with intracellular H protein was less than that of CD4^+^ cells. There was little or no detectable intracellular H protein in either CD4^+^ or CD8^+^ cells from Ad-H vaccinated animals which had also received GMCSF. The annexin V-positive cells were minimal in all animals.

## Discussion

In light of the recent success of the rinderpest eradication campaign, PPRV would be an excellent candidate for eradication [44,45] and moves have already been made towards this. The availability of a DIVA vaccine would facilitate PPRV sero-surveillance programmes and speed up the steps leading to disease eradication [46]. In countries newly affected by PPRV, where sporadic outbreaks of disease occur and where the disease is not endemic, a DIVA vaccine would be of value to prevent animal movement restrictions being imposed on countries which cannot prove that animals have been vaccinated and not infected. Since the virus has only 6 genes, all of which are essential for growth, creating a DIVA version of the current live PPRV vaccines would require expressing an extra protein from the viral genome (positively marked vaccine). The alternative is to express one or two viral proteins from an alternative virus vector, thereby eliciting immune protection while not inducing the complete repertoire of antibodies induced following natural infection or vaccination with a live, attenuated PPRV vaccine. This was done successfully for RPV using vaccinia or capripox virus as the vaccine vectors [47-50]. However, these constructs were never used in the field, in part because they did not offer the same duration of protection as the existing vaccine and because the rinderpest eradication campaign was completed without an explicit requirement for a DIVA vaccine. MVA expressing PPRV F and H proteins have been shown to protect goats against subsequent challenge with virulent PPR but two doses of vaccine were given prior to challenge [51], which would not be practical for a small ruminant vaccination programme. Capripox virus vectors expressing PPRV glycoproteins have been developed [4,52]. However, in one case, the ability of the vaccine to protect against PPRV was not investigated [52], while in the other, although protection against PPRV was proven [4], the vaccine has not been used in the field. The positive aspects of using a recombinant capripox are that it would simultaneously vaccinate against two serious sheep/goat diseases; in addition, capripox-based vaccines would benefit from the intrinsic thermotolerance of poxviruses. However, recombinant capripox vaccines may not be suitable as PPRV DIVA vaccines, as vaccinated animals did not all give good antibody responses, possibly due to pre-existing vector immunity. This is important as it is the comparative antibody response that is likely to be used as the DIVA test, with infected animals having anti-PPRV N and anti-PPRV H antibodies, while the vaccinees will only have anti-PPRV H antibodies. There are existing, well established and validated commercially available cELISAs for both anti-N and anti-H antibodies, making this an attractive DIVA test.

Despite their successful use in several trials as vectors for human vaccines, the FP-based vaccines elicited very poor antibody responses in goats, as well as poor cell-mediated immune responses. The low level of responses in small ruminants may be due to apoptosis of FP virus-infected antigen presenting cells in these animals, as has been shown recently in cattle [37]. Because of the low level of immune response to the PPRV proteins expressed from the FP vectors, we did not pursue these constructs through to challenge, since they would not be useful DIVA vaccines even if effective in protecting the vaccinated animals from PPRV.

Replication-deficient adenovirus-vectored vaccines induce potent CD8^+^ and CD4^+^ T-cell responses as well as high antibody responses [53], and appear to be safe [54]. Furthermore, the Ad vector also acts as an adjuvant [55]. Adjuvant effects in experimental vaccines have been demonstrated by co-expressing cytokines such as IL-2, IL-12 and GMCSF [56], presenting other options for vaccine formulation. One of the drawbacks of Ad5-vectored vaccines in humans has been that most people have previously been infected with this virus, and the pre-existing antibodies can inhibit the efficacy of the vaccine [57]. However, vaccines based on Ad5 may be suitable for use in livestock since these animals will not have pre-existing immunity to the vector. Large scale production of Ad viruses can be achieved [58] and, furthermore, Ad viruses can be made more thermostable and efficacious in a range of formulations that further promote stability [59,60].

Vaccination of goats with Ad-H, alone or with a similar dose of Ad-F, induced levels of H-specific, neutralising antibodies within 2 to 3 weeks that were comparable to those induced by live, attenuated PPRV vaccines [40,61]. Furthermore, these antibodies were maintained for several months following vaccination. While this work was in progress, similar Ad constructs were reported [62], which induced a similar level of neutralising antibody as that described in the present study, following a single dose of replication defective Ad-H. In another recent study, replication competent canine adenovirus expressing PPRV H was also found to be effective at eliciting neutralising antibody in goats [63]. Unfortunately, neither of these studies went on to determine the ability of the Ad-vectored vaccines to protect against virulent PPRV. This is important, as the critical elements of the immune response required for protection against PPRV are not yet known. While the current live attenuated PPRV vaccine induces neutralising antibody, and a titre > 1:10 is used as a marker for competency of preparations of such vaccines, attenuated morbillivirus vaccines also induce cell-mediated immunity [64,65], which may also be important in protection. We have demonstrated that vaccination with Ad-H, or Ad-H and Ad-F, induced a potent effector memory CD8^+^ response in goats, although the number of H-specific CD8^+^ IFNγ^+^ cells had declined to basal levels by 15 weeks post-vaccination. Further studies are therefore needed to determine the effect of Ad-H vaccination on persistence of central memory CD8^+^ T cells in goats. No detailed studies have been carried out to establish the mechanisms of protection induced by live, attenuated PPRV vaccines. However, studies on rinderpest showed that induction of neutralising antibodies by vaccination with purified viral proteins did not protect against infection [66], suggesting that it is not possible to deduce protection based on antibody alone. We therefore analysed the ability of Ad-vectored vaccines to protect against infection with virulent PPRV, and demonstrated that a single dose of Ad expressing the PPRV H protein can protect against PPRV challenge up to 4 months after vaccination. Furthermore, vaccinated goats did not appear to excrete infectious virus from the nasopharynx, suggesting that they may not transmit virus to susceptible, unvaccinated animals. This is the first time that an Ad-vectored vaccine has been shown to protect against virulent PPRV challenge, and shows that the immune responses elicited by the replication-defective vaccine are sufficient to protect the vaccinated animal from infection, with a protective response that is sustained for at least 4 months. Longer term studies will be required to determine the duration of PPRV H-specific serum antibodies induced by Ad-H.

In the study by Wang et al. [62], it was suggested that co-expression of F and H proteins induced higher levels of neutralising antibody than vaccination with Ad expressing either F or H alone. This is a similar finding to that seen in cattle vaccinated with vaccinia virus expressing RPV H, F or both H and F, where it was suggested that the combination of H and F induced stronger protection against RPV [67]. In our studies, expression of H alone (experiment 3) induced neutralising antibody titres at least as high as those seen in animals vaccinated with Ad-F plus Ad-H (experiment 2). This may be because we gave a higher dose of Ad than that used by Wang et al. [62], and the strength of the response to H alone, coupled with the adjuvant effect of Ad, dominated any co-operative effect of vaccination with Ad H and F together.

This is the first time that the effect of virulent PPRV infection on specific immune cell sub-sets has been analyzed. We have shown that whereas the proportion of circulating WC1^+^ γ/δ T-cells and CD14^+^ monocyte/macrophage cells did not change after PPRV infection of control goats, there was a decrease in the proportion of circulating CD4^+^ cells 4 days after challenge. This decrease may have been due, at least in part, to infection with PPRV, as the proportion of CD4^+^ cells staining for intracellular H was greater than that of CD8^+^ cells, 4 days after PPRV challenge. The reduction in CD4^+^ cells was not observed in any of the Ad-H vaccinated animals. There was a slight increase in the percentage of CD8^+^ T-cells at 7 days pc in all animals, suggesting induction of CTL responses by PPRV infection. Co-administration of Ad-GMCSF at the time of vaccination appeared to have an effect on infection of CD4^+^ and CD8^+^ cells with PPRV. These animals had lower levels of detectable intracellular viral H protein in both the live CD4^+^ and live CD8^+^ T cells 4 days after challenge, compared with the other vaccine groups.

The contribution of GMCSF and IL-2 in boosting immune responses to Ad vaccination is not clear from this study. However, the results from the 2^nd^ experiment suggested that the combination of Ad-IL-2 and Ad-GMCSF induced higher H-specific serum antibodies and a greater H-specific CD8^+^ IFNγ^+^ response compared with Ad-F and Ad-H alone. The level of N-specific antibodies after challenge with PPRV was significantly lower in the two vaccine groups that received Ad-IL-2 compared with the other groups of goats, suggesting that there was less replication of the challenge virus in these animals and, therefore, that co-administration of Ad-IL-2 induced a more effective protective immune response, even though a significant effect on T cell responses, H-specific or neutralising antibody levels was not seen. It will be interesting to determine if an adjuvant effect of the co-expressed cytokines is more obvious at lower doses of Ad-H/Ad-F. If such studies demonstrate that a co-administered Ad-vectored cytokine has a dose sparing effect on an Ad-vectored PPRV vaccine, then it may be possible to construct a recombinant Ad, which can have an insert of ~7.5 kb, expressing both PPRV H and cytokine.

In conclusion, we have demonstrated that a single vaccination with a recombinant Ad expressing the PPRV H protein induced PPRV-specific neutralising antibodies, primed CD8^+^ T cells, was safe, and completely protected goats against PPR for at least 4 months.

## Competing interests

The authors declare that they have no competing interests.

## Authors’ contributions

BH carried out the experimental animal studies, cellular immunoassays, and drafted the manuscript. JB carried out the serological and virological assays. CB undertook the qRT-PCR to determine levels of virus. MB and GT conceived of the study, participated in its design and coordination, participated in the experimental animal studies, and helped to draft the manuscript. All authors read and approved the final manuscript.

## References

[B1] Sinnathamby G Renukaradhya GJ Rajasekhar M Nayak R Shaila MS Immune responses in goats to recombinant hemagglutinin-neuraminidase glycoprotein of Peste des petits ruminants virus: identification of a T cell determinant Vaccine 2001 19 4816 4823 10.1016/S0264-410X(01)00210-9 11535334

[B2] Jones L Giavedoni L Saliki JT Brown C Mebus C Yilma T Protection of goats against peste des petits ruminants with a vaccinia virus double recombinant expressing the F and H genes of rinderpest virus Vaccine 1993 11 961 964 10.1016/0264-410X(93)90386-C 8212844

[B3] Romero CH Barrett T Kitching RP Bostock C Black DN Protection of goats against peste des petits ruminants with recombinant capripoxviruses expressing the fusion and haemagglutinin protein genes of rinderpest virus Vaccine 1995 13 36 40 10.1016/0264-410X(95)80008-2 7762275

[B4] Diallo A Minet C Berhe G Le Goff C Black DN Fleming M Barrett T Grillet C Libeau G Goat immune response to capripox vaccine expressing the hemagglutinin protein of peste des petits ruminants Ann N Y Acad Sci 2002 969 88 91 10.1111/j.1749-6632.2002.tb04356.x 12381569

[B5] Wild F Giraudon P Spehner D Drillien R Lecocq JP Fowlpox virus recombinant encoding the measles-virus fusion protein - protection of mice against fatal measles encephalitis Vaccine 1990 8 441 442 10.1016/0264-410X(90)90243-F 2174596

[B6] Alvarez-Lajonchere L Amador-Cañizares Y Frias R Milian Y Musacchio A Guerra I Acosta-Rivero N Martinez G Castro J Puentes P Cosme K Dueñas-Carrera S Immunization with a recombinant fowlpox virus expressing a hepatitis C virus core-E1 polyprotein variant, protects mice and African green monkeys (Chlorocebus aethiops sabaeus) against challenge with a surrogate vaccinia virus Biotechnol Appl Biochem 2008 51 97 105 10.1042/BA20070182 18215116

[B7] Lasaro MO Haut LH Zhou X Xiang Z Zhou D Li Y Giles-Davis W Li H Engram JC Dimenna LJ Bian A Sazanovich M Parzych EM Kurupati R Small JC Wu TL Leskowitz RM Klatt NR Brenchley JM Garber DA Lewis M Ratcliffe SJ Betts MR Silvestri G Ertl HC Vaccine-induced T cells provide partial protection against high-dose rectal SIVmac239 challenge of rhesus macaques Mol Ther 2011 19 417 426 10.1038/mt.2010.238 21081905 PMC3034846

[B8] Reyes-Sandoval A Sridhar S Berthoud T Moore AC Harty JT Gilbert SC Gao G Ertl HC Wilson JC Hill AV Single-dose immunogenicity and protective efficacy of simian adenoviral vectors against Plasmodium berghei Eur J Immunol 2008 38 732 741 10.1002/eji.200737672 18266272

[B9] Bassett JD Swift SL Bramson JL Optimizing vaccine-induced CD8(+) T-cell immunity: focus on recombinant adenovirus vectors Expet Rev Vaccine 2011 10 1307 1319 10.1586/erv.11.88 21919620

[B10] Yang TC Dayball K Wan YH Bramson J Detailed analysis of the CD8+ T-Cell response following adenovirus vaccination J Virol 2003 77 13407 13411 10.1128/JVI.77.24.13407-13411.2003 14645597 PMC296052

[B11] Yang TC Millar J Groves T Grinshtein N Parsons R Takenaka S Wan Y Bramson JL The CD8+ T cell population elicited by recombinant adenovirus displays a novel partially exhausted phenotype associated with prolonged antigen presentation that nonetheless provides long-term immunity J Immunol 2006 176 200 210 16365411 10.4049/jimmunol.176.1.200

[B12] Tatsis N Fitzgerald JC Reyes-Sandoval A Harris-McCoy KC Hensley SE Zhou D Lin SW Bian A Xiang ZQ Iparraguirre A Lopez-Camacho C Wherry EJ Ertl HC Adenoviral vectors persist in vivo and maintain activated CD8+ T cells: implications for their use as vaccines Blood 2007 110 1916 1923 10.1182/blood-2007-02-062117 17510320 PMC1976365

[B13] Kim S Jang JE Yu JR Chang J Single mucosal immunization of recombinant adenovirus-based vaccine expressing F1 protein fragment induces protective mucosal immunity against respiratory syncytial virus infection Vaccine 2010 28 3801 3808 10.1016/j.vaccine.2010.03.032 20362203

[B14] Lee LN Baban D Ronan EO Ragoussis J Beverley PC Tchilian EZ Chemokine gene expression in lung CD8 T cells correlates with protective immunity in mice immunized intra-nasally with Adenovirus-85A BMC Med Genom 2010 3 46 10.1186/1755-8794-3-46 PMC2967494 20942964

[B15] Lobanova LM Baig TT Tikoo SK Zakhartchouk AN Mucosal adenovirus-vectored vaccine for measles Vaccine 2010 28 7613 7619 10.1016/j.vaccine.2010.09.055 20887832

[B16] Min L Mohammad Isa SA Shuai W Piang CB Nih FW Kotaka M Ruedl C Cutting edge: granulocyte-macrophage colony-stimulating factor is the major CD8+ T cell-derived licensing factor for dendritic cell activation J Immunol 2010 184 4625 4629 10.4049/jimmunol.0903873 20357255

[B17] Bowne WB Wolchok JD Hawkins WG Srinivasan R Gregor P Blachere NE Moroi Y Engelhorn ME Houghton AN Lewis JJ Injection of DNA encoding granulocyte-macrophage colony-stimulating factor recruits dendritic cells for immune adjuvant effects Cytokines Cell Mol Ther 1999 5 217 225 10850386

[B18] Lai L Kwa S Kozlowski PA Montefiori DC Ferrari G Johnson WE Hirsch V Villinger F Chennareddi L Earl PL Moss B Amara RR Robinson HL Prevention of infection by a granulocyte-macrophage colony-stimulating factor co-expressing DNA/modified vaccinia Ankara simian immunodeficiency virus vaccine J Infect Dis 2011 204 164 173 10.1093/infdis/jir199 21628671 PMC3143670

[B19] Robinson HL Montefiori DC Villinger F Robinson JE Sharma S Wyatt LS Earl PL McClure HM Moss B Amara RR Studies on GM-CSF DNA as an adjuvant for neutralizing Ab elicited by a DNA/MVA immunodeficiency virus vaccine Virology 2006 352 285 294 10.1016/j.virol.2006.02.011 16740288

[B20] Mahdavi M Ebtekar M Khorram Khorshid HR Azadmanesh K Hartoonian C Hassan ZM ELISPOT analysis of a new CTL based DNA vaccine for HIV-1 using GM-CSF in DNA prime/peptide boost strategy: GM-CSF induced long-lived memory responses Immunol Lett 2011 140 14 20 10.1016/j.imlet.2011.05.005 21679728

[B21] Kadir Z Ma X Li J Zhang F Granulocyte-macrophage colony-stimulating factor enhances the humoral immune responses of mouse zona pellucida 3 vaccine strategy based on DNA and protein coadministration in BALB/c mice Reprod Sci 2013 20 400 407 10.1177/1933719112459236 23111125 PMC4077514

[B22] Reali E Canter D Zeytin H Schlom J Greiner JW Comparative studies of Avipox-GM-CSF versus recombinant GM-CSF protein as immune adjuvants with different vaccine platforms Vaccine 2005 23 2909 2921 10.1016/j.vaccine.2004.11.060 15780740

[B23] Schell JB Bahl K Rose NF Buonocore L Hunter M Marx PA LaBranche CC Montefiori DC Rose JK Viral vectored granulocyte-macrophage colony stimulating factor inhibits vaccine protection in an SIV challenge model: protection correlates with neutralizing antibody Vaccine 2012 30 4233 4239 10.1016/j.vaccine.2012.04.046 22537983 PMC3367070

[B24] Zheng Q Fan D Gao N Chen H Wang J Ming Y Li J An J Evaluation of a DNA vaccine candidate expressing prM-E-NS1 antigens of dengue virus serotype 1 with or without granulocyte-macrophage colony-stimulating factor (GM-CSF) in immunogenicity and protection Vaccine 2011 29 763 771 10.1016/j.vaccine.2010.11.014 21095256

[B25] Chen H Gao N Fan D Wu J Zhu J Li J Wang J Chen Y An J Suppressive effects on the immune response and protective immunity to a JEV DNA vaccine by co-administration of a GM-CSF-expressing plasmid in mice PLoS One 2012 7 e34602 10.1371/journal.pone.0034602 22493704 PMC3321030

[B26] Caligiuri MA Murray C Robertson MJ Wang E Cochran K Cameron C Schow P Ross ME Klumpp TR Soiffer RJ Selective modulation of human natural killer cells in vivo after prolonged infusion of low dose recombinant interleukin 2 J Clin Invest 1993 91 123 132 10.1172/JCI116161 7678599 PMC330005

[B27] Storset AK Berntsen G Larsen HJ Kinetics of IL-2 receptor expression on lymphocyte subsets from goats infected with Mycobacterium avium subsp. paratuberculosis after specific in vitro stimulation Vet Immunol Immunopathol 2000 77 43 54 10.1016/S0165-2427(00)00227-0 11068065

[B28] Li J Liang X Huang Y Meng S Xie R Deng R Yu L Enhancement of the immunogenicity of DNA vaccine against infectious bursal disease virus by co-delivery with plasmid encoding chicken interleukin 2 Virology 2004 329 89 100 10.1016/j.virol.2004.07.033 15476877

[B29] Premenko-Lanier M Rota PA Rhodes G Verhoeven D Barouch DH Lerche NW Letvin NL Bellini WJ McChesney MB DNA vaccination of infants in the presence of maternal antibody: a measles model in the primate Virology 2003 307 67 75 10.1016/S0042-6822(02)00036-3 12667815

[B30] Wong HT Cheng SC Sin FW Chan EW Sheng ZT Xie Y A DNA vaccine against foot-and-mouth disease elicits an immune response in swine which is enhanced by co-administration with interleukin-2 Vaccine 2002 20 2641 2647 10.1016/S0264-410X(02)00212-8 12034088

[B31] Toubaji A Hill S Terabe M Qian J Floyd T Simpson RM Berzofsky JA Khleif SN The combination of GM-CSF and IL-2 as local adjuvant shows synergy in enhancing peptide vaccines and provides long term tumor protection Vaccine 2007 25 5882 5891 10.1016/j.vaccine.2007.05.040 17602804

[B32] Chow YH Chiang BL Lee YL Chi WK Lin WC Chen YT Tao MH Development of Th1 and Th2 populations and the nature of immune responses to hepatitis B virus DNA vaccines can be modulated by codelivery of various cytokine genes J Immunol 1998 160 1320 1329 9570550

[B33] Ahlers JD Dunlop N Alling DW Nara PL Berzofsky JA Cytokine-in-adjuvant steering of the immune response phenotype to HIV-1 vaccine constructs: granulocyte-macrophage colony-stimulating factor and TNF-alpha synergize with IL-12 to enhance induction of cytotoxic T lymphocytes J Immunol 1997 158 3947 3958 9103465

[B34] Xu R Megati S Roopchand V Luckay A Masood A Garcia-Hand D Rosati M Weiner DB Felber BK Pavlakis GN Sidhu MK Eldridge JH Egan MA Comparative ability of various plasmid-based cytokines and chemokines to adjuvant the activity of HIV plasmid DNA vaccines Vaccine 2008 26 4819 4829 10.1016/j.vaccine.2008.06.103 18657584 PMC9036319

[B35] Nobiron I Thompson I Brownlie J Collins ME Cytokine adjuvancy of BVDV DNA vaccine enhances both humoral and cellular immune responses in mice Vaccine 2001 19 4226 4235 10.1016/S0264-410X(01)00157-8 11457549

[B36] Zhang C Wang B Wang M GM-CSF and IL-2 as adjuvant enhance the immune effect of protein vaccine against foot-and-mouth disease Virol J 2011 8 7 10.1186/1743-422X-8-7 21214955 PMC3024958

[B37] Cubillos-Zapata C Guzman E Turner A Gilbert SC Prentice H Hope JC Charleston B Differential effects of viral vectors on migratory afferent lymph dendritic cells in vitro predict enhanced immunogenicity in vivo J Virol 2011 85 9385 9394 10.1128/JVI.05127-11 21752909 PMC3165747

[B38] Perez de Val B Vidal E Villarreal-Ramos B Gilbert SC Andaluz A Moll X Martin M Nofrarias M McShane H Vordermeier HM Domingo M A multi-antigenic adenoviral-vectored vaccine improves BCG-induced protection of goats against pulmonary tuberculosis infection and prevents disease progression PLoS One 2013 8 e81317 10.1371/journal.pone.0081317 24278420 PMC3836889

[B39] Skinner MA Laidlaw SM Eldaghayes I Kaiser P Cottingham MG Fowlpox virus as a recombinant vaccine vector for use in mammals and poultry Expert Rev Vaccine 2005 4 63 76 10.1586/14760584.4.1.63 15757474

[B40] Diallo A Taylor WP Lefèvre PC Provost A Attenuation of a strain of rinderpest virus: potential homologous live vaccine Rev Elev Med Vet Pays Trop 1989 42 311 319 (in French) 2485537

[B41] Das SC Baron MD Barrett T Recovery and characterization of a chimeric rinderpest virus with the glycoproteins of peste-des-petits-ruminants virus: homologous F and H proteins are required for virus viability J Virol 2000 74 9039 9047 10.1128/JVI.74.19.9039-9047.2000 10982348 PMC102100

[B42] Webster DP Dunachie S McConkey S Poulton I Moore AC Walther M Laidlaw SM Peto T Skinner MA Gilbert SC Hill AVS Safety of recombinant fowlpox strain FP9 and modified vaccinia virus Ankara vaccines against liver-stage P. falciparum malaria in non-immune volunteers Vaccine 2006 24 3026 3034 10.1016/j.vaccine.2005.10.058 16488059

[B43] Batten CA Banyard AC King DP Henstock MR Edwards L Sanders A Buczkowski H Oura CC Barrett T A real time RT-PCR assay for the specific detection of Peste des petits ruminants virus J Virol Methods 2011 171 401 404 10.1016/j.jviromet.2010.11.022 21126540

[B44] Albina E Kwiatek O Minet C Lancelot R Servan de Almeida R Libeau G Peste des Petits Ruminants, the next eradicated animal disease? Vet Microbiol 2013 165 38 44 10.1016/j.vetmic.2012.12.013 23313537

[B45] Baron MD Parida S Oura CA Peste des petits ruminants: a suitable candidate for eradication? Vet Rec 2011 169 16 21 10.1136/vr.d3947 21724765

[B46] Diallo A Minet C Le Goff C Berhe G Albina E Libeau G Barrett T The threat of peste des petits ruminants: progress in vaccine development for disease control Vaccine 2007 25 5591 5597 10.1016/j.vaccine.2007.02.013 17399862

[B47] Romero CH Barrett T Chamberlain RW Kitching RP Fleming M Black DN Recombinant capripoxvirus expressing the hemagglutinin protein gene of rinderpest virus: protection of cattle against rinderpest and lumpy skin disease viruses Virology 1994 204 425 429 10.1006/viro.1994.1548 8091673

[B48] Yilma T Hsu D Jones L Owens S Grubman M Mebus C Yamanaka M Dale B Protection of cattle against rinderpest with vaccinia virus recombinants expressing the HA or F gene Science 1988 242 1058 1061 10.1126/science.3194758 3194758

[B49] Belsham GJ Anderson EC Murray PK Anderson J Barrett T Immune response and protection of cattle and pigs generated by a vaccinia virus recombinant expressing the F protein of rinderpest virus Vet Rec 1989 124 655 658 10.1136/vr.124.25.655 2763430

[B50] Yamanouchi K Inui K Sugimoto M Asano K Nishimaki F Kitching RP Takamatsu H Barrett T Immunisation of cattle with a recombinant vaccinia vector expressing the haemagglutinin gene of rinderpest virus Vet Rec 1993 132 152 156 10.1136/vr.132.7.152 8456545

[B51] Chandran D Reddy KB Vijayan SP Sugumar P Rani GS Kumar PS Rajendra L Srinivasan VA MVA recombinants expressing the fusion and hemagglutinin genes of PPRV protects goats against virulent challenge Indian J Microbiol 2010 50 266 274 10.1007/s12088-010-0026-9 23100840 PMC3450065

[B52] Chen WY Hu S Qu LM Hu QQ Zhang QA Zhi HB Huang KH Bu ZG A goat poxvirus-vectored peste-des-petits-ruminants vaccine induces long-lasting neutralization antibody to high levels in goats and sheep Vaccine 2010 28 4742 4750 10.1016/j.vaccine.2010.04.102 20471441

[B53] Kaufman DR Liu J Carville A Mansfield KG Havenga MJ Goudsmit J Barouch DH Trafficking of antigen-specific CD8+ T lymphocytes to mucosal surfaces following intramuscular vaccination J Immunol 2008 181 4188 4198 18768876 10.4049/jimmunol.181.6.4188 PMC2580672

[B54] Deal C Pekosz A Ketner G Prospects for oral replicating adenovirus-vectored vaccines Vaccine 2013 31 3236 3243 10.1016/j.vaccine.2013.05.016 23707160 PMC3750733

[B55] Geutskens SB van der Eb MM Plomp AC Jonges LE Cramer SJ Ensink NG Kuppen PJ Hoeben RC Recombinant adenoviral vectors have adjuvant activity and stimulate T cell responses against tumor cells Gene Ther 2000 7 1410 1416 10.1038/sj.gt.3301251 10981668

[B56] Abaitua F Rodriguez JR Garzon A Rodriguez D Esteban M Improving recombinant MVA immune responses: potentiation of the immune responses to HIV-1 with MVA and DNA vectors expressing Env and the cytokines IL-12 and IFN-gamma Virus Res 2006 116 11 20 10.1016/j.virusres.2005.08.008 16214252

[B57] Thacker EE Timares L Matthews QL Strategies to overcome host immunity to adenovirus vectors in vaccine development Expert Rev Vaccine 2009 8 761 777 10.1586/erv.09.29 PMC3700409 19485756

[B58] Ferreira TB Ferreira AL Carrondo MJ Alves PM Effect of re-feed strategies and non-ammoniagenic medium on adenovirus production at high cell densities J Biotechnol 2005 119 272 280 10.1016/j.jbiotec.2005.03.009 15885836

[B59] Cruz PE Silva AC Roldao A Carmo M Carrondo MJ Alves PM Screening of novel excipients for improving the stability of retroviral and adenoviral vectors Biotechnol Prog 2006 22 568 576 10.1021/bp050294y 16599578

[B60] Lameiro MH Malpique R Silva AC Alves PM Melo E Encapsulation of adenoviral vectors into chitosan-bile salt microparticles for mucosal vaccination J Biotechnol 2006 126 152 162 10.1016/j.jbiotec.2006.04.030 16757053

[B61] Saravanan P Sen A Balamurugan V Rajak KK Bhanuprakash V Palaniswami KS Nachimuthu K Thangavelu A Dhinakarraj G Hegde R Singh RK Comparative efficacy of peste des petits ruminants (PPR) vaccines Biologicals 2010 38 479 485 10.1016/j.biologicals.2010.02.003 20199873

[B62] Wang Y Liu G Chen Z Li C Shi L Li W Huang H Tao C Cheng C Xu B Li G Recombinant adenovirus expressing F and H fusion proteins of peste des petits ruminants virus induces both humoral and cell-mediated immune responses in goats Vet Immunol Immunopathol 2013 154 1 7 10.1016/j.vetimm.2013.05.002 23707075

[B63] Qin J Huang H Ruan Y Hou X Yang S Wang C Huang G Wang T Feng N Gao Y Xia X A novel recombinant Peste des petits ruminants-canine adenovirus vaccine elicits long-lasting neutralizing antibody response against PPR in goats PLoS One 2012 7 e37170 10.1371/journal.pone.0037170 22623990 PMC3356378

[B64] Lund BT Tiwari A Galbraith S Baron MD Morrison WI Barrett T Vaccination of cattle with attenuated rinderpest virus stimulates CD4(+) T cell responses with broad viral antigen specificity J Gen Virol 2000 81 2137 2146 10950969 10.1099/0022-1317-81-9-2137

[B65] Gans HA Yasukawa LL Sung P Sullivan B DeHovitz R Audet S Beeler J Arvin AM Measles humoral and cell-mediated immunity in children aged 5-10 years after primary measles immunization administered at 6 or 9 months of age J Infect Dis 2013 207 574 582 10.1093/infdis/jis719 23300162 PMC3549597

[B66] Bassiri M Ahmad S Giavedoni L Jones L Saliki JT Mebus C Yilma T Immunological responses of mice and cattle to baculovirus-expressed F and H proteins of rinderpest virus: lack of protection in the presence of neutralizing antibody J Virol 1993 67 1255 1261 8437215 10.1128/jvi.67.3.1255-1261.1993 PMC237491

[B67] Verardi PH Aziz FH Ahmad S Jones LA Beyene B Ngotho RN Wamwayi HM Yesus MG Egziabher BG Yilma TD Long-term sterilizing immunity to rinderpest in cattle vaccinated with a recombinant vaccinia virus expressing high levels of the fusion and hemagglutinin glycoproteins J Virol 2002 76 484 491 10.1128/JVI.76.2.484-491.2002 11752138 PMC136817

